# Supplementary material for: Immediate Adverse Events Following COVID-19 Vaccination in Australian Pharmacies: A Retrospective Review
Source: Vaccines (Basel). 2022 Nov 29;10(12):2041. doi: 10.3390/vaccines10122041 (PMC9787804; doi:10.3390/vaccines10122041)
Supplement: Supplementary file 1 [file vaccines-10-02041-s001.zip › Table S1 - MedDRA Terms by Brand and Dose.pdf]

Table S1. Number of I-AEFI identified in free text by brand and dose of COVID-19 vaccine

	COVID-19 Vaccine													
	AstraZeneca Dose		Moderna Dose				Novavax Dose		Pfizer Dose				Pfizer Paediatric Dose	
	1	2	1	2	3	4	1	2	1	2	3	4	1	2
Angina pectoris	0	0	1	1	0	0	0	0	0	0	0	0	0	0
Anxiety	2	1	0	1	0	0	0	0	0	0	0	0	0	0
Asthma	0	0	1	0	0	0	0	0	0	0	0	0	0	0
Cardiac flutter	0	0	1	0	0	0	0	0	0	0	0	0	0	0
Chest pain	1	0	0	1	0	0	0	0	0	0	0	0	0	0
Cold sweat	0	0	0	0	0	0	0	0	1	0	0	0	0	0
Confusional state	1	0	0	0	0	0	0	0	0	0	0	0	0	0
Cyanosis	0	0	0	0	0	0	0	0	0	0	0	1	0	0
Dysgeusia	2	0	0	0	0	1	0	0	0	0	0	0	0	0
Dyspepsia	0	0	1	0	0	0	0	0	0	0	0	0	0	0
Dysphonia	1	0	0	0	0	0	0	0	0	0	0	0	0	0
Dyspnoea	0	0	1	0	0	0	0	0	0	1	0	0	0	0
Dystonia	0	0	0	0	0	0	0	0	0	1	0	0	0	0
Erythema	1	0	0	0	0	0	0	0	0	0	0	0	0	0
Fatigue	0	0	2	0	0	2	0	0	0	0	0	0	0	0
Feeling abnormal	0	0	1	0	0	0	0	0	0	0	0	0	0	0
Feeling hot	1	0	2	1	0	0	0	0	0	1	0	0	0	0
Foaming at mouth	1	0	0	0	0	0	0	0	0	0	0	0	0	0
Headache	0	0	2	1	0	0	0	0	0	0	0	0	0	0
Heart rate decreased	1	0	0	0	0	0	0	0	0	0	0	0	0	0
Hot flush	1	0	0	0	0	0	0	0	0	0	0	0	0	0
Hyperhidrosis	10	0	3	3	0	2	0	0	1	0	0	1	0	0
Hypertension	0	0	2	0	0	0	0	0	0	0	0	0	0	0
Hyperventilation	0	0	1	0	0	0	0	0	0	0	0	0	0	0
Hypoaesthesia	1	1	1	0	0	0	0	0	0	0	0	0	0	0
Hypoaesthesia oral	0	0	1	0	0	0	0	0	0	0	0	0	0	0
Hypotension	0	0	3	0	0	0	0	0	1	0	0	0	0	0
Influenza like illness	3	0	0	1	0	1	0	0	0	0	0	0	0	0
Injection site bruising	0	0	0	0	0	1	0	0	0	0	0	1	0	0
Injection site haemorrhage	8	2	5	1	0	1	0	0	0	0	0	0	0	0

Injection site mass	1	0	0	1	0	1	0	0	0	0	0	1	0	0
Lethargy	1	1	0	0	0	0	0	0	0	0	0	0	0	0
Limb discomfort	0	0	0	0	0	0	0	1	0	0	0	0	0	0
Malaise	1	1	2	1	0	1	0	0	0	0	0	0	0	0
Muscle spasms	0	0	2	1	0	0	0	0	0	0	0	0	0	0
Muscle tightness	0	0	1	0	0	0	0	0	0	0	0	0	0	0
Muscle twitching	0	1	0	0	0	0	0	0	0	0	0	0	0	0
Muscular weakness	0	0	1	0	0	0	0	0	0	0	0	0	0	0
Myalgia	1	0	0	0	0	0	0	0	0	0	0	0	0	0
Neuralgia	0	0	0	1	0	0	0	0	0	0	0	0	0	0
Orthostatic hypotension	0	0	1	0	0	0	0	0	0	0	0	0	0	0
Pain in extremity	1	0	4	1	0	0	0	0	0	0	0	2	0	0
Palpitations	1	0	0	0	0	1	0	0	0	0	0	0	0	0
Paraesthesia	5	1	3	2	0	1	0	0	2	0	0	0	0	0
Paraesthesia oral	0	0	1	1	0	1	0	0	0	0	0	0	0	0
Pharyngeal hypoaesthesia	0	0	0	0	0	1	0	0	0	0	0	0	0	0
Pruritus	0	0	1	0	0	0	0	0	0	0	0	0	0	0
Pyrexia	0	0	0	2	0	1	0	0	0	0	0	0	0	0
Rash	1	0	1	1	0	0	0	1	0	0	0	1	0	0
Respiratory arrest	1	0	0	0	0	0	0	0	0	0	0	0	0	0
Sensation of foreign body	1	0	0	0	0	0	0	0	0	0	0	0	0	0
Swelling	0	0	0	0	0	1	0	0	0	0	0	0	0	0
Swollen tongue	0	1	0	0	0	0	0	0	0	0	0	0	0	0
Tachycardia	1	0	2	1	0	0	0	0	0	0	1	1	0	0
Tinnitus	1	0	3	0	0	0	0	0	0	0	0	0	0	0
Tremor	5	0	3	0	0	0	0	0	0	0	0	1	0	0
Urticaria	0	0	0	0	0	1	0	0	0	0	0	0	0	0
Vaccination site rash	0	0	2	2	0	1	0	0	0	0	0	0	0	0
Vision blurred	2	0	3	0	0	0	0	0	0	0	0	0	0	0
