# Supplementary material for: Immediate Adverse Events Following COVID-19 Vaccination in Australian Pharmacies: A Retrospective Review
Source: Vaccines (Basel). 2022 Nov 29;10(12):2041. doi: 10.3390/vaccines10122041 (PMC9787804; doi:10.3390/vaccines10122041)
Supplement: Supplementary file 1 [file vaccines-10-02041-s001.zip › Table S2 - Management.pdf]

Table S2. Management of MedDRA mapped reactions by pre-defined management and other management.

	AD	AMB	LD	SM	PM	W	S	SS	AH	E	Info	TGA	Cold	Lift legs	Juice	BP	BPM	BSL	Para	FU EOD	Sit	O2	ºC	MB	Pred	TP	A-I
Anxiety	0	1	2	0	1	0	0	1	0	0	0	0	0	0	0	1	1	0	0	0	0	0	0	2	0	0	0
Asthma	0	0	0	1	1	1	0	0	0	0	0	0	0	0	0	0	0	0	0	0	1	0	0	1	0	0	0
Cardiac flutter	0	0	0	1	0	0	0	0	0	0	0	0	0	0	0	0	0	0	0	0	0	0	0	0	0	0	0
Chest pain	0	1	0	1	1	0	0	0	0	0	0	0	0	0	0	1	0	0	0	0	0	0	0	1	0	0	0
Cold sweat	0	0	1	0	0	0	0	0	0	0	0	0	0	0	0	0	0	0	0	0	0	0	0	0	0	0	0
Confusional state	0	1	1	0	0	0	1	0	0	0	0	0	0	0	0	0	0	1	0	1	0	0	0	0	0	0	0
Cyanosis	0	0	1	0	0	1	0	0	0	0	0	0	0	1	0	0	0	0	0	0	0	0	0	0	0	0	0
Dysgeusia	0	0	1	2	1	0	0	0	0	0	1	0	0	0	0	0	0	0	0	0	0	0	0	0	0	0	0
Dyspepsia	0	0	0	1	0	1	0	0	0	0	0	0	0	0	0	0	0	0	0	0	1	0	0	0	0	0	0
Dysphonia	0	1	0	0	0	0	0	0	0	0	0	0	0	0	0	0	0	0	0	0	0	0	0	0	0	0	0
Dyspnoea	1	2	2	0	0	0	0	0	0	0	0	0	1	0	0	0	0	0	0	0	0	0	0	0	0	0	0
Dystonia	0	0	1	0	1	0	0	1	0	0	0	0	0	0	0	0	1	0	0	0	0	0	0	1	0	0	0
Erythema	0	0	0	0	0	0	0	0	1	0	0	0	0	0	0	0	0	0	0	0	1	0	0	0	0	0	0
Fatigue	0	0	0	0	0	0	0	0	0	0	0	0	0	0	0	0	0	0	0	0	0	0	1	0	0	0	0
Feeling abnormal	0	0	0	0	1	0	0	0	1	0	0	0	0	0	0	0	0	0	0	0	0	0	0	0	0	0	0
Feeling hot	0	1	0	2	4	1	0	0	0	2	0	0	0	0	0	0	0	0	0	0	2	0	0	1	0	0	0
Foaming at mouth	0	0	1	0	1	1	1	0	0	0	0	0	0	0	0	0	0	0	0	0	0	0	0	0	0	0	0
Headache	0	2	2	0	0	1	0	0	0	0	1	0	0	0	0	0	0	0	1	0	0	0	0	0	0	0	0
Heart rate decreased	0	0	0	0	1	0	0	0	0	0	0	0	0	0	0	1	1	0	0	0	1	0	0	0	0	0	0
Hot flush	0	0	0	0	0	1	0	0	0	0	0	0	0	0	0	1	1	0	0	0	0	0	0	0	0	0	0
Hyperhidrosis	0	3	9	1	3	8	4	0	0	0	0	0	2	0	0	0	0	0	1	1	2	0	0	1	0	0	0
Hypertension	0	1	1	0	0	1	0	0	0	0	0	0	0	0	0	1	1	0	0	0	0	0	0	0	0	0	0
Hyperventilation	0	1	0	0	0	0	0	0	0	0	0	0	0	0	0	0	0	0	0	0	0	0	0	0	0	0	0
Hypoaesthesia	0	0	0	0	2	0	0	0	0	0	0	0	0	0	0	0	0	0	0	0	0	0	0	0	0	0	0
Hypoaesthesia oral	0	0	1	0	1	1	0	0	0	0	0	0	0	0	0	1	1	0	0	0	0	1	0	0	0	0	0
Hypotension	0	0	2	0	1	2	2	0	0	0	0	0	0	0	0	2	2	0	0	0	1	0	0	0	0	0	0
Influenza like illness	0	0	1	1	0	0	0	0	0	1	0	0	0	0	0	0	0	0	1	0	0	0	0	1	0	0	1

Injection site bruising	0	0	0	0	1	0	0	0	0	0	0	0	1	0	0	0	0	0	0	0	0	0	0	0	0	0	0
Injection site haemorrhage	0	0	0	0	1	0	0	0	0	0	0	0	0	0	0	0	0	0	0	0	0	0	0	0	0	0	0
Injection site mass	0	0	0	1	2	0	0	0	1	0	0	0	2	0	0	0	0	0	0	0	0	0	0	0	0	0	0
Lethargy	0	0	0	0	1	1	0	0	0	0	0	0	0	0	0	0	0	0	0	0	1	0	0	0	0	0	0
Limb discomfort	0	0	0	0	1	0	0	0	0	0	0	0	0	0	0	0	0	0	0	0	0	0	0	0	0	0	0
Malaise	0	1	5	0	1	0	1	0	0	0	0	0	1	1	0	1	0	0	0	0	1	0	0	0	0	0	0
Muscle spasms	0	2	3	0	0	0	0	0	0	0	0	0	0	0	0	1	0	0	0	0	0	0	0	0	0	0	0
Muscle tightness	0	0	0	0	1	1	0	0	0	0	0	0	0	0	0	0	0	0	0	0	0	0	0	0	0	0	0
Muscle twitching	0	0	1	0	0	0	0	0	0	0	0	0	0	0	0	0	0	0	0	0	0	0	0	0	0	0	0
Muscular weakness	0	0	0	0	0	0	0	0	0	0	0	0	0	0	0	0	1	0	0	0	0	0	0	1	0	0	0
Myalgia	0	0	0	0	0	0	0	0	0	0	0	0	0	0	0	0	0	0	1	0	0	0	0	0	0	0	1
Neuralgia	0	0	0	1	0	1	0	0	0	0	0	0	0	0	0	0	0	0	0	0	0	0	0	0	0	0	0
Orthostatic hypotension	0	0	0	0	0	1	0	0	0	0	0	0	0	0	0	0	0	0	1	0	1	0	0	0	0	0	0
Pain in extremity	0	2	1	1	0	0	0	0	1	0	0	0	1	0	0	1	1	0	1	0	0	0	0	0	0	0	3
Palpitations	0	1	1	0	1	1	0	0	0	0	0	0	0	0	0	0	1	0	0	1	0	0	0	0	0	0	0
Paraesthesia	0	2	7	2	8	3	2	0	0	0	0	0	1	1	1	3	2	0	1	0	2	0	0	0	0	0	0
Paraesthesia oral	1	0	1	0	1	0	0	0	1	0	0	0	0	0	0	0	0	0	0	0	0	0	0	0	0	0	0
Pharyngeal hypoaesthesia	0	0	0	0	0	0	0	0	0	0	0	0	0	0	0	0	0	0	0	0	0	0	0	1	0	0	0
Pruritus	1	0	1	1	0	1	0	0	0	0	0	0	0	0	0	0	0	0	0	0	0	0	0	0	0	0	0
Pyrexia	0	0	0	0	0	1	0	0	0	0	0	0	0	0	0	0	0	0	1	0	0	0	1	0	0	1	1
Rash	0	0	0	0	0	0	0	0	3	0	0	0	2	0	0	0	0	0	1	0	0	0	0	0	0	1	0
Respiratory arrest	1	1	1	0	0	0	0	0	0	0	0	0	0	0	0	0	0	0	0	0	0	0	0	0	0	0	0
Sensation of foreign body	0	0	0	0	1	0	0	0	0	0	0	0	0	0	0	0	0	0	0	0	0	0	0	0	0	0	0
Swelling	0	0	0	0	0	0	0	0	0	0	0	0	1	0	0	0	0	0	1	0	0	0	0	0	0	0	0
Swollen tongue	0	0	0	0	0	1	0	0	1	0	0	0	0	0	0	1	1	0	0	0	1	0	0	0	0	0	0
Tachycardia	0	3	4	0	0	1	1	0	0	0	0	0	0	0	0	0	2	0	0	0	1	0	0	0	0	0	0
Tinnitus	0	0	4	0	0	1	0	0	0	0	0	1	1	0	0	0	0	0	0	0	0	0	0	0	0	0	0
Tremor	1	6	8	1	2	3	1	1	0	0	0	0	1	1	0	1	0	0	0	0	0	0	0	0	0	0	0
Urticaria	0	0	0	0	0	0	0	0	1	0	0	0	1	0	0	0	0	0	0	0	0	0	0	0	0	0	0

Vaccination site rash	0	0	0	0	1	0	0	0	3	0	0	0	0	0	0	0	0	0	0	0	0	0	0	0	0	1	0
Vision blurred	0	1	2	0	2	2	0	0	0	0	0	0	0	0	0	1	1	0	0	0	0	0	0	0	0	0	0

AD—administered adrenaline; AMB—called ambulance; LD—laid patient down; SM—self-monitor at home for worsening symptoms; PM—monitor in pharmacy until symptoms resolve; W—give glass of water; S—give sugar (glucose, lollipop, chocolate); SS—give salbutamol via spacer; AH—give antihistamine; E—give electrolytes; Info—provide government information on vaccine or consumer medicines information; TGA—report to Therapeutic Goods Administration; Cold—give cold compress/cold pack; Lift legs—elevated legs; Juice— give patient juice; BP—checked blood pressure; BPM—check heart rate/pulse; BSL—checked blood sugar level; Para—give paracetamol; FU EOD—follow-up with patient at the end of the day; Sit—sit patient down; O2—check oxygen saturation; ºC—check temperature; MB—monitor breathing; Pred—advised to take prednisolone; TP—give topical preparation; A-I—give anti-inflammatory
