# Supplementary figures and images for: Immediate Adverse Events Following COVID-19 Vaccination in Australian Pharmacies: A Retrospective Review
Source: Vaccines (Basel). 2022 Nov 29;10(12):2041. doi: 10.3390/vaccines10122041 (PMC9787804; doi:10.3390/vaccines10122041)

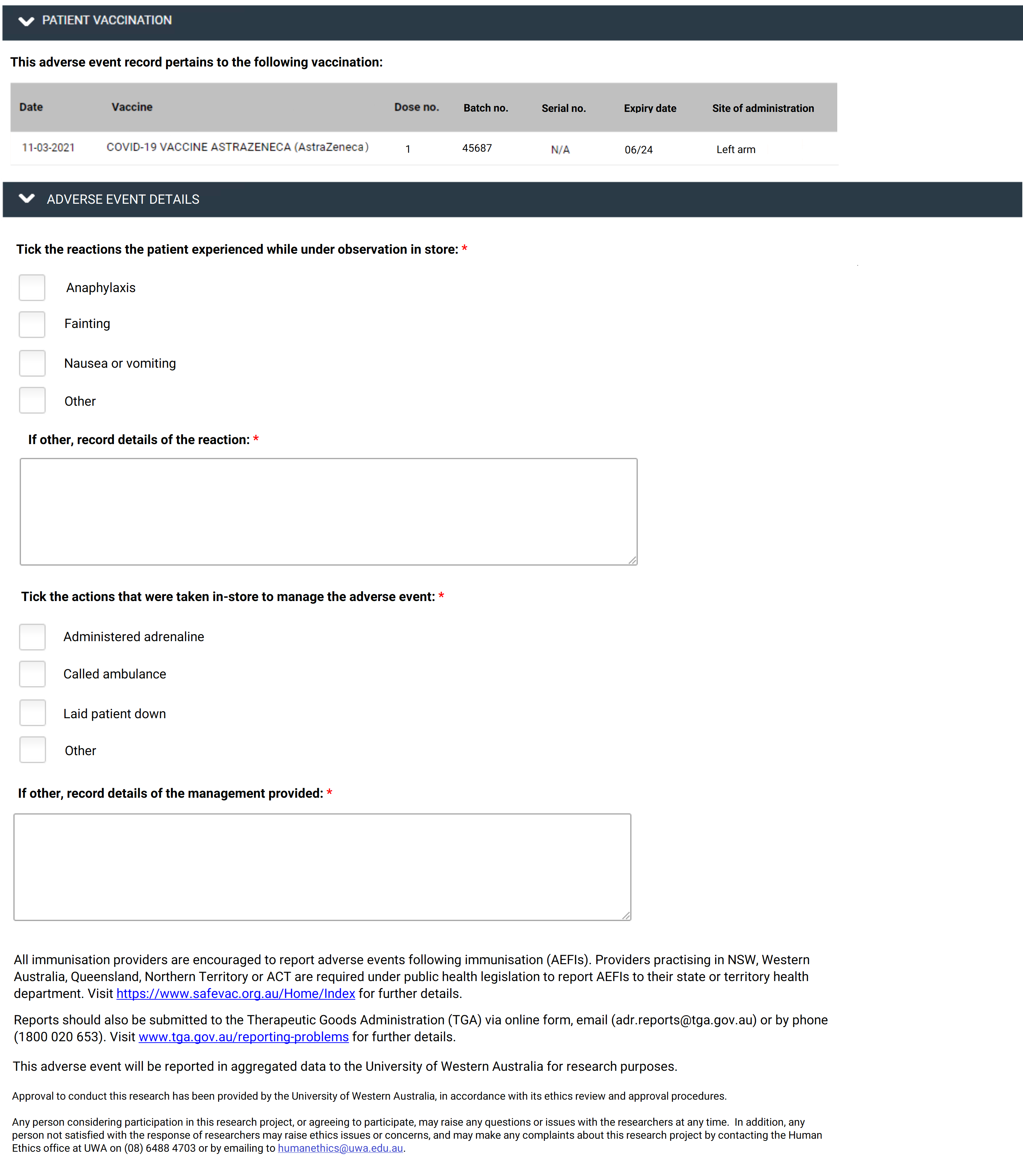

Supplement: Supplementary file 1 [file vaccines-10-02041-s001.zip › Figure S1 - IAEFI Recording Form.png]
